# Role of microRNA-146a in normal and malignant hematopoietic stem cell function

**DOI:** 10.3389/fgene.2014.00219

**Published:** 2014-07-09

**Authors:** Jimmy L. Zhao, Daniel T. Starczynowski

**Affiliations:** ^1^Division of Biology and Biological Engineering, California Institute of TechnologyPasadena, CA, USA; ^2^David Geffen School of Medicine, University of CaliforniaLos Angeles, CA, USA; ^3^Division of Experimental Hematology and Cancer Biology, Cincinnati Children’s Hospital Medical CenterCincinnati, OH, USA; ^4^Department of Cancer Biology, University of CincinnatiCincinnati, OH, USA

**Keywords:** microRNA, hematopoietic stem cells, NF-kappa B, myelodysplastic syndromes, immune system

## Abstract

Regulation of hematopoiesis is controlled by microRNAs (miRNAs). In this review, we focus on miR-146a, and its role in regulating normal and malignant hematopoiesis. miR-146a is a negative regulator of immune cell activation by repressing two targets, TRAF6 and IRAK1. Genetic deletion of miR-146a confirmed a role of miR-146a during innate immune signaling as well as for hematopoietic stem cell function. miR-146a is also implicated in the pathogenesis of human myelodysplastic syndromes (MDSs) as it is located within a commonly deleted region on chromosome 5, and miR-146a-deficient mice exhibit features of an MDS-like disease. With new insight into miR-146a through genetic and expression analyses, we highlight and discuss the recent advances in the understanding of miR-146a in physiological hematopoiesis during steady-state and inflammation, as well as in MDS.

## INTRODUCTION

Mammalian hematopoiesis is a highly regulated process involving multipotent stem and progenitor cells giving rise to all blood cell types. During steady-state, hematopoiesis is in a homeostatic balance to ensure that newly differentiated cells replenish dying blood cells. At the same time, hematopoiesis is a highly dynamic process that can respond efficiently to external stimuli, such as during inflammation and infections. Under inflammatory or infectious states, such as part of the host innate immune response, hematopoietic stem and progenitor cells (HSPCs) respond by increasing the production of mature blood cells, particularly immune cells of the myeloid lineage (Baldridge et al., 2011). Regulation of hematopoiesis is tightly controlled by transcription factors, chromatin remodeling factors, and small non-coding RNAs, such as microRNAs (miRNAs; Chen et al., 2004; O’Connell et al., 2010; O’Connell and Baltimore, 2012). Herein, we will focus on one particular miRNA, miR-146a, and its role in regulating normal and malignant hematopoiesis. The importance of miR-146a became apparent almost 10 years ago through a microarray screen in search of miRNAs that are regulated by NF-κB transcription factors (Taganov et al., 2006). Through target analysis, it became evident that miR-146a may be an important negative regulator of immune cell activation by repressing two targets, TRAF6 and IRAK1, both of which are signaling transducers upstream of NF-κB. A mouse model with targeted genetic deletion of miR-146a confirmed a role of miR-146a during innate immune signaling as well as for hematopoietic stem cell (HSC) function (Boldin et al., 2011; Zhao et al., 2011). miR-146a is also implicated in the pathogenesis of human myelodysplastic syndromes (MDSs) as it is located within a commonly deleted region on chromosome 5 (Starczynowski et al., 2010). In this review, we highlight some of the recent advances in the understanding of miR-146a in physiological hematopoiesis during steady-state and inflammation, as well as its dysregulation in malignant hematopoiesis, especially in MDS.

## DISCOVERY OF THE miR-146 FAMILY

miR-146a and miR-146b are two miRNAs of the same family. They have an identical seed region and putative mRNA targets, but differ in their mature strand sequence by only two nucleotides and in their stem-loop secondary structure. In humans, miR-146a resides on chromosome 5q33.3 and miR-146b resides on chromosome 10q24.32, while in mice, miR-146a resides on chromosome 11 and miR-146b on chromosome 19. The miR-146 family was initially described as NF-κB target genes through a microarray study to identify miRNAs that were upregulated upon lipopolysaccharide (LPS) stimulation in THP1 cells (Taganov et al., 2006). Three miRNAs, miR-146, miR-155 and miR-132, showed significantly increased expression following LPS simulation. Furthermore, a promoter analysis identified two functional and conserved NF-κB binding sites upstream of the miR-146a gene. This finding identified the first NF-κB-regulated miRNA family.

## EXPRESSION OF miR-146a IN NORMAL AND MALIGNANT HEMATOPOIETIC CELLS

**FIGURE 1:**
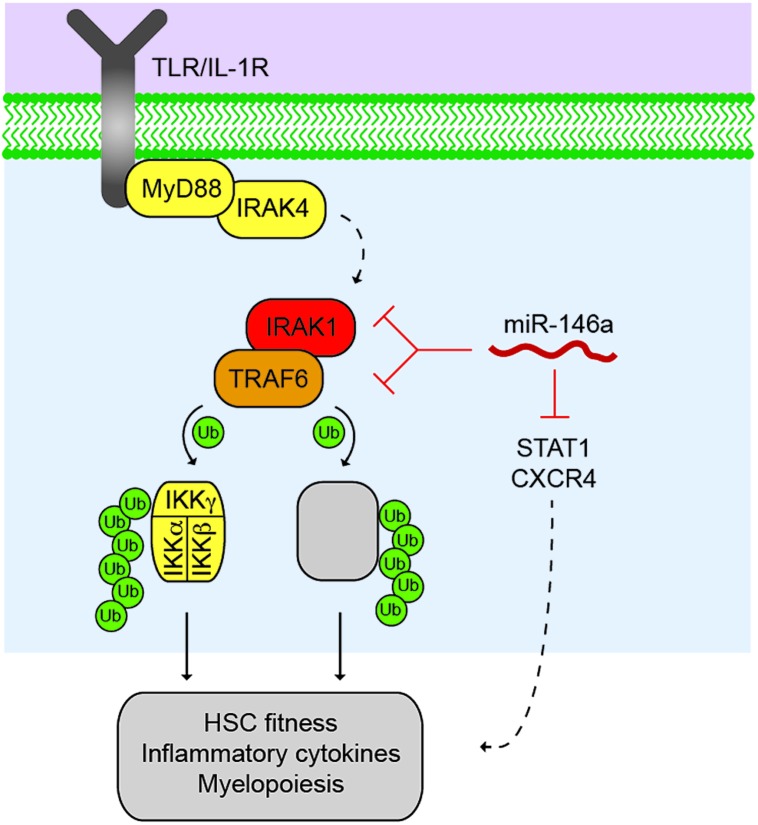
**Control of the TRAF6 signalosome by miR-146a.** Activated Toll-like receptor (TLR) or lnterleukin-1 receptor (IL1R) results in the recruitment of MyD88 and IRAK4, which activates the serine/threonine kinase, IRAKI, through IRAK4-dependent phosphorylation (dashed line). IRAKI associates with an E3 ubiquitin ligase, TRAF6, which mediates the activation of the IKK complex through K63-linked polyubiquitin chains, resulting in NF-κB transcription factor activation. TRAF6 also regulates other proteins (as indicated by grey box) that may also contribute to immune signaling and malignancies. miR-146a suppresses IRAKI and TRAF6 protein expression through direct binding at 3′UTR sites within IRAKI and TRAF6 mRNAs. Reduced expression and/or deletion of miR-146a results in derepression of IRAKI and TRAF6 protein, and increased downstream pathway activation resulting in reduced HSC fitness, increased inflammatory cytokine expression, and altered myeloid differentiation. miR-146a also represses STAT1 and CXCR4 in hematopoietic cells, which may also contribute to the HSC defects following deletion of miR-146a.

The expression pattern of miR-146a and miR-146b overlaps within certain mouse hematopoietic lineages. Their expression is similarly high in double-positive and double-negative thymocytes and primitive bone marrow (BM) cells (i.e., lineage negative BM cells) by >2-fold as compared to LSK cells (lineage negative/Sca1+/cKit+). Within primitive BM cells, their expression is higher (~1.5 fold) in HSCs and in myeloid progenitors compared to other progenitor cell subtypes. In contrast, miR-146a and miR-146b expression differs in mature myeloid (Mac1+), erythroid (Ter119+), and lymphoid (CD4+ and CD8+) cells. Although the expression of miR-146a is detectable in HSCs and throughout mature blood cell maturation, its expression increases >5-fold as HSCs mature, suggesting a potential role throughout hematopoietic development (Starczynowski et al., 2011; Zhao et al., 2013). In general, the basal expression of miR-146a is modest, except in certain specialized myeloid cell lineages, including Ly-6Clow monocytes and epidermal Langerhans cells, which have constitutively high levels of expression (Jurkin et al., 2010; Etzrodt et al., 2012). However, miR-146a expression can be highly induced in hematopoietic cells by a wide range of infectious and inflammatory stimuli, including Toll-like receptor (TLR) ligands, pro-inflammatory cytokines, T-cell receptor ligands, as well as numerous pathogens, implicating a role of miR-146a (**Figure 1**) in immune cell activation and stress-mediated hematopoiesis (So et al., 2013). The basal and inducible expression pattern of miR-146a is regulated by a combination of lineage-specific transcription factors, including PU.1 and c-ETS (Cameron et al., 2008; Curtale et al., 2010; Ghani et al., 2011), and activation-dependent transcription factors, most notably NF-κB and AP1 (Taganov et al., 2006; Ho et al., 2014). In non-hematopoietic cells, miR-146a can be upregulated by the transcription factor Snail in colorectal cancer stem cells (Hwang et al., 2014), and miR-146b is directly induced by transcription factor STAT3 in breast epithelial cancer cells (Xiang et al., 2014). The expression of miR-146a or miR-146b has not been as extensively studied in human hematopoietic cells. However, consistent with the mouse studies, miR-146a is expressed at high levels in human BM CD34+ HSPC cells (Starczynowski et al., 2010).

miR-146a is located within the commonly deleted region associated with del(5q) MDS (or 5q-syndrome). MDS are heterogeneous HSC disorders characterized by persistent cytopenia due to ineffective hematopoiesis and dysplasia of BM cells. As the disease progresses, patients may develop BM failure. MDS patients are also at an elevated risk of transforming to acute myeloid leukemia (AML; Scott and Deeg, 2011). Despite the heterogeneous clinical presentation and variable outcome of MDS, many patients have shared underlying pathological features. These include peripheral blood cytopenia affecting one or more blood lineages (e.g., erythrocytes, granulocytes, monocytes, and/or megakaryocytes), and normal/hypercellular marrows with morphological dysplasia (Tefferi and Vardiman, 2009). As per the diagnostic criteria, blasts account for less than 30% of the marrow. Examination of miR-146a in BM HSPC from del(5q) MDS patients has revealed reduced expression by approximately half, consistent with deletion of a single allele (Starczynowski et al., 2010; Votavova et al., 2011; **Table 1**). In addition, miR-146a is consistently downregulated in a large non-del(5q) MDS cohort suggesting that multiple mechanisms may contribute to its reduced expression in MDS (**Table 1**; Sokol et al., 2011; Zhao et al., 2013). In this non-del(5q) MDS cohort, miR-146a consists of a miRNA diagnostic signature that can distinguish MDS patients from age-matched controls (Sokol et al., 2011). This finding is further confirmed through another large cohort of unselected MDS BM samples showing that miR-146a is down regulated ~5-fold in MDS patients as compared to healthy controls; in contrast, such down regulation is not consistently observed in AML BM cells (Zhao et al., 2013). Although miR-146a is consistently down regulated in del(5q) MDS, additional miRNAs residing on chr 5q (i.e., miR-145) may also contribute to aspects of MDS pathogenesis.

**Table 1:** Evidence of low miR-146a expression in MDS.

Study	Cell source	Experimental group	Control group	Experimental method
Sokol (2011)	BM MNC	Low or INT-1	Normal	microarray
Starczynowski (2010)	BM MNC	del(5q)	Normal and MDS dip(5q)	qRT-PCR
Votavova (2011)	CD34+	del(5q)	Normal	qRT-PCR
Zhao (2013)	BM MNC	MDS	Normal	qRT-PCR

*BM, bone marrow; MNC, mononuclear cells.*

## ROLE OF miR-146a DURING STEADY-STATE HEMATOPOIESIS

miR-146a germline knockout (miR-146a–/–) mice are born at the expected Mendelian frequency and show no obvious abnormalities at a young age. Furthermore, miR-146a does not appear to be essential for fetal and steady-state hematopoiesis in young mice. All hematopoietic cell subset frequencies, from primitive HSCs to mature myeloid and lymphoid populations, are identical in young (~6 week old) miR-146a–/– mice as compared to wild-type (WT) mice. In addition, the ability of miR-146a–/– long-term HSCs to generate all hematopoietic lineages competitively with WT HSCs in lethally irradiated recipient mice is nearly identical for up to the first 10 months post-transplant. However, after 10 months, miR-146a–/– HSCs are out-numbered by co-transplanted WT HSCs (Zhao et al., 2013). The subtle cell intrinsic defect seen only in miR-146a–/– long-term HSCs after 10 months suggests an interesting role of miR-146a in maintaining the self-renewal of long-term HSCs.

The impact of enforced expression of miR-146a on hematopoiesis is not as dramatic as miR-146a deletion. Overall, most studies have shown a minor impact of miR-146a overexpression on HSPC numbers and functions (Opalinska et al., 2010; Ghani et al., 2011; Starczynowski et al., 2011). It remains unclear whether enforced expression of miR-146a would impair myelopoiesis under stressed conditions. A recent study has generated a transgenic mouse model with constitutive miR-146a overexpression via a non-targeted insertion of a lentiviral vector containing mouse miR-146a sequence under an ubiquitin promoter (Guo et al., 2013). This miR-146a transgenic mouse develops spontaneous immune pathologies starting at 3 weeks of age that are characterized by enlarged spleen and lymph nodes, inflammatory cell infiltration of liver and lungs and enhanced expansion of T and B cells. The study suggests that the lymphoproliferative and autoimmune pathologies seen in this mouse model are due to miR-146a-mediated repression of Fas expression in germinal center B cells. This interesting and rather surprising result is not what would have been predicted based on miR-146a genetic knockout and knockdown studies, which have all shown that miR-146a is an important negative regulator of cell activation and inflammation in immune and non-hematopoietic cells (Cameron et al., 2008; Perry et al., 2008; Hou et al., 2009; Chassin et al., 2010; Lu et al., 2010; Nakasa et al., 2011; Yang et al., 2012). In addition, several previous studies have shown that miR-146a overexpression in mouse BM transplant models has minimal and at best transient effects on hematopoiesis. However, there are two major differences between the transgenic model and BM transplant models: the distribution of tissue expression and timing of expression. The transgenic model overexpresses miR-146a in all tissues, including non-hematopoietic cells, and during embryonic development. Whether overexpression of miR-146a in non-hematopoietic tissues and/or enforced expression of miR-146a during fetal hematopoiesis contribute to the discrepancy is not known. In summary, miR-146a is not essential for hematopoietic cell development, as miR-146a-deficiency does not have major effects on steady-state hematopoiesis in young mice; however, miR-146a may have a role in the self-renewal of long-term HSCs. On the other hand, enforced expression of miR-146a in hematopoietic cells also appears to have minimal impact on adult steady-state hematopoiesis.

## ROLE OF miR-146a DURING STRESSED HEMATOPOIESIS

In contrast to steady-state hematopoiesis, miR-146a plays a significant role in regulating hematopoiesis during stressed conditions. Nominal stressors, such as natural aging and repeated low level of inflammatory stimulation, have produced striking phenotypes in miR-146a–/– mice. This is best illustrated in a series of aging studies conducted by examining matched cohorts of WT and miR-146a–/– mice over a 2 year period in standard pathogen-free conditions (Boldin et al., 2011; Zhao et al., 2011, 2013). At 2 months of age, miR-146a–/– mice exhibit normal hematopoietic development; however, at 4 months, miR-146a–/– mice develop a transient hypercellular state in the BM accompanied with increased number of HSCs and mature cells, reflecting a state of increased HSC proliferation and differentiation. By 8 months, miR-146a–/– mice become severely depleted of BM cells, while spleens become enlarged with predominant myelopoiesis. This process of BM depletion and splenic myelopoiesis continues until mice succumb to BM failure and/or pathologic myeloid-related diseases. For miR-146a–/– mice beyond 1 year of age, they begin to exhibit accelerated mortality from a myeloproliferative disease, peripheral pancytopenia, and ultimately myeloid or lymphoid cancers.

The decline of HSC function is observed in younger mice and prior to declines in HSC number, as HSCs from four-month-old miR-146a–/– mice already exhibit a differentiation defect, as compared to WT HSCs in competitive repopulation studies. Interestingly, the long-term aging phenotypes can be recapitulated in young miR-146a–/– mice through serial injection of sublethal doses of LPS (Zhao et al., 2013). Based on these observations, we propose that the aging process provides opportunities for an organism to encounter a variety of inflammatory stimuli, including environmental pathogens, commensal bacteria and endogenous inflammatory mediators. Normally, these inflammatory stimuli are short-lived and stimulate only a transient burst of HSC proliferation and myeloid production. The resolution of the immune reaction is mediated in part by the upregulation of miR-146a. However, in the absence of miR-146a, the inflammatory signal is prolonged, leading to excessive HSC activation and myelopoiesis. Furthermore, repeated exposure to unregulated inflammatory stimuli ultimately leads to premature exhaustion of HSCs and sustained pathological myelopoiesis.

It was recently shown that HSPCs have an incredible ability to release a wide range of cytokines in response to TLR stimulation. Not surprisingly, miR-146a–/– HSPCs, namely short-term HSCs and multipotent progenitor cells (MPPs), produce increased amount of pro-inflammatory cytokines, most notably IL-6, TNF-α, GM-CSF and IL-1β, upon TLR stimulation (Zhao et al., 2014). In addition to the cell-intrinsic function within HSPCs, miR-146a also impacts hematopoiesis through the regulation of cytokine production in monocytes, macrophages and effector T cells. Furthermore, miR-146a is shown to function in a number of non-hematopoietic tissues, including endothelial cells, gastrointestinal and lung epithelial cells (Perry et al., 2008; Chassin et al., 2010; Cheng et al., 2013). Given that non-hematopoietic environments contribute to pathologic hematopoiesis, especially tumor induction, seen in miR-146a–/– mice (Zhao et al., 2013), it would be prudent to examine whether miR-146a regulates the HSC BM niche. In addition to hematopoietic development, miR-146a has an indispensable role in the immune system as a negative regulator of mature immune cell activation. The involvement of miR-146a in the host immune response against infections and autoimmune diseases has also been extensively studied. This has been comprehensively reviewed elsewhere recently (Chan et al., 2013; Montagner et al., 2013; So et al., 2013) and will not be covered here.

## ROLE OF miR-146a AND INNATE IMMUNE SIGNALING IN MDS

The first evidence that miR-146a deficiency may contribute to hematopoietic defects associated with MDS was shown in mice with reduced levels of miR-146a, and a neighboring miRNA (miR-145). Knockdown of miR-145 and miR-146a using a miRNA decoy approach in mouse HSPC resulted in hematological abnormalities including elevated platelets, neutropenia, megakaryocytic dysplasia, and myeloid leukemia. The distinction between miR-145 and miR-146a’s contribution to the hematopoietic defects has been revealed by examination of the miR-146a-deficient mice. As described above, knockout of miR-146a results in an early onset of myeloid expansion in the BM, and progression to more aggressive diseases such as lymphomas, BM failure, and myeloid leukemia (Lu et al., 2010; Boldin et al., 2011; Zhao et al., 2011). That the miR-146a knockout mice do not show evidence of elevated platelets suggests that loss of miR-145 may contribute to thrombocytosis associated with del(5q) MDS patients (Kumar et al., 2011).

At the molecular level, the signaling transducer, TRAF6, is a well-characterized target of miR-146a. TRAF6 expression is de-repressed in miR-146a–/– HSPCs, macrophages, T cells and B cells (Boldin et al., 2011; Yang et al., 2012; Zhao et al., 2013). In the regulation of hematopoiesis, miR-146a is shown to function through a defined signaling pathway involving TRAF6, NF-κB, and IL-6 (Zhao et al., 2013). Overexpression of TRAF6 in mouse HSPC using a retroviral approach mimicked some of the hematopoietic defects observed in mice with loss of miR-146a. Enforced TRAF6 expression also resulted in elevated platelets, neutropenia, dysplasia, and myeloid leukemia in a subset of mice. Some of the effects are mediated by IL-6 as overexpression of TRAF6 in IL-6-deficient HSPC restored platelets and neutrophil counts. However, the IL6-deficiency did not delay BM failure and AML. Despite similarities between miR-146a-deficient and TRAF6-transduced HSPC, the level of TRAF6 expression in transduced HSPC is at least 10-fold higher than observed in miR-146a-deficient HSPC. To better understand the contribution of TRAF6 to the miR-146a-deficient HSPC phenotype, the enforced expression levels of TRAF6 in BM transplant mice need to reflect what is observed in miR-146a-deficient HSPC and in MDS patients.

## MOLECULAR CONSEQUENCES OF miR-146a DELETION

Multiple groups have now validated TRAF6 and IRAK1 as key targets of miR-146a (Taganov et al., 2006; Hou et al., 2009; Starczynowski et al., 2010; Zhao et al., 2011; Yang et al., 2012; Rhyasen et al., 2013; So et al., 2013). The TRAF6 3′ untranslated region (UTR) has two or three highly conserved miR-146a binding sites (Taganov et al., 2006; Starczynowski et al., 2010), while the IRAK1 3′UTR has two highly conserved miR-146a binding sites. When luciferase is fused to the TRAF6 or IRAK1 3′ UTR, overexpression of miR-146a results in reduced luciferase activity (but not when fused to a 3′ UTR with a mutant miR-146a binding site), indicating direct binding of miR-146a to the TRAF6 and IRAK1 3′ UTR (Taganov et al., 2006; Starczynowski et al., 2010). In mouse HSC cells, overexpression of miR-146a results in reduced endogenous TRAF6 and IRAK1 protein, and conversely, knockdown of miR-146a results in derepression of TRAF6 and IRAK1 protein (Starczynowski et al., 2010, 2011). As highlighted above, strong evidence that miR-146a regulates TRAF6 protein expression comes from miR-146a knockout mice (Boldin et al., 2011; Zhao et al., 2011). These mice have increased TRAF6 and IRAK1 (2-fold) expression within the hematopoietic compartment. Importantly, in MDS/AML patients, TRAF6 and IRAK1 protein levels are inversely correlated with miR-146a expression.

As a transcription factor downstream of TRAF6/IRAK1, NF-κB activation can be detected in miR-146a-deficient monocytes/macrophages, effector T cells and HSCs by gene expression, biochemical and NF-κB-GFP reporter mouse studies (Boldin et al., 2011; Etzrodt et al., 2012; Yang et al., 2012; Zhao et al., 2013; Ho et al., 2014). Although miR-146a appears to have a cell-intrinsic role in regulating both the long-term HSC self-renewal and HSPC proliferation, the molecular consequences of miR-146a expression as they relate to HSC function are still not fully understood. The canonical NF-κB pathway involving subunits p65 and p50 is thought to contribute to some of the defects observed in the hematopoietic system of miR-146a-deficient mice following derepression of TRAF6, as genetic deletion of the p50 subunit attenuates some of the pathologies in the absence of miR-146a. Non-canonical NF-κB signaling, as well as additional signaling pathways may also contribute to aspects of hematopoiesis regulated by miR-146a (Etzrodt et al., 2012). The signaling networks regulated by miR-146a become further complicated as TRAF6 regulates additional signaling pathways through its E3 ubiquitin ligase domain. As such, future studies to address the molecular mechanisms controlled by TRAF6 overexpression in miR-146a-deficient HSPC need to be performed.

Although, TRAF6 and IRAK1 remain as key targets regulated by miR-146a in the hematopoietic system, other molecules and their associated signaling pathways have emerged as direct targets of miR-146a (**Figure 1**). Among them, STAT1 is shown to be a direct target of miR-146a in regulatory and effector T cells (Lu et al., 2010; Wang et al., 2013). We have also found STAT1 to be de-repressed in miR-146a-deficient HSPCs (Zhao et al., 2013). CXCR4 may also be a direct target of miR-146a in megakaryopoiesis (Labbaye et al., 2008). Several groups have identified a number of interesting targets of miR-146a in non-hematopoietic cells, including HuR in endothelial cells (Cheng et al., 2013) and Numb in gastrointestinal cells and melanoma cells (Ghorpade et al., 2013; Forloni et al., 2014; Hwang et al., 2014).

## CONCLUSION AND FUTURE DIRECTIONS

It is established that miR-146a is a critical and indispensable regulator of inflammatory hematopoiesis and immune cell activation by repressing two key targets, TRAF6 and IRAK1, and in part by regulating NF-κB activation. Furthermore, there is no doubt that miR-146a-deficiency is involved in the pathogenesis of several human immune-mediated diseases and hematologic malignancies, in particular MDS. Recently, three independent groups identified Numb as an important target of miR-146a in different cellular and disease contexts, opening up a new direction for functional and mechanistic investigation of miR-146a. Given the importance and complex functions of miR-146a in different tissues, there is a need to further dissect the function of miR-146a, the molecular consequences of aberrant TRAF6 and/or IRAK1 derepression, and validation of other associated mRNA targets in a cell type-and context-specific manner through the study of conditional miR-146a knockout mice. There is also a large knowledge gap in the function of miR-146b that shares an identical seed sequence and differs in only two nucleotides within the mature sequence. The knowledge on whether re-expressing or re-introducing miR-146a has therapeutic benefit in hematologic diseases will be an important step in advancing therapies for diseases with diminished miR-146a expression. Lastly, inhibiting the derepressed targets of miR-146a, as shown for IRAK1 in MDS (Rhyasen et al., 2013), constitutes another attractive and feasible therapeutic approach.

## Conflict of Interest Statement

The authors declare that the research was conducted in the absence of any commercial or financial relationships that could be construed as a potential conflict of interest.
